# Cone beam imaging and audiometric evaluation of the eustachian tube in OSMF: A case-control study

**DOI:** 10.6026/973206300223556

**Published:** 2026-06-30

**Authors:** Reema Manoj, Mandavi Waghmare, Boddu Tejvanti, Richa Kamble, Iqra Malgundkar, Gargie Gharat

**Affiliations:** 1 Department of Oral Medicine and Radiology, DY Patil University, School of Dentistry, Navi Mumbai, Maharashtra, India; 2 Department of Dentistry, DY Patil University, School of Dentistry, Navi Mumbai, Maharashtra, India

**Keywords:** Hearing loss, cone beam computer tomography (CBCT), eustachian tube (ET), oral submucous fibrosis (OSMF)

## Abstract

The rising incidence of oral submucous fibrosis (OSMF) among younger Indians and its potentially aggressive progression necessitate investigation of deeper structural changes, including those affecting the eustachian tube (ET). Hence, this 2-year departmental study evaluated morphometric and volumetric alterations in 77 participants (48 OSMF patients and 29 healthy controls) using clinical examination, CBCT and audiometric assessment, with OSMF cases classified by functional stage. Half of the OSMF patients showed an interincisal opening of 25-35 mm (M2) and significant reductions in ET length and volume were observed in the study group compared with controls (p<0.05). ET dimensions and audiometric findings also differed significantly across OSMF stages (M1-M4), indicating progressive structural compromise associated with worsening auditory function (p<0.05). Thus, we report CBCT-based evidence that OSMF is associated with stage-related ET morphometric and volumetric reduction, contributing to auditory impairment and broadening understanding of its deeper oropharyngeal effects.

## Background:

Oral submucous fibrosis (OSMF) is a chronic potentially malignant disorder marked by fibrosis of the oral submucous, most common in Asian populations, especially in the Indian subcontinent, Southeast Asia and parts of Africa [1]. Its etiology is multifactorial, with contributors such as betel quid and areca nut use, spicy foods, genetic susceptibility and nutritional deficiencies, all promoting fibroblast proliferation, collagen deposition and loss of tissue elasticity [2]. OSMF prevalence continues to rise in developing countries, affecting about 0.5% of the population [3]. Increasing incidence among younger Indians is particularly concerning, as early-onset disease may progress more aggressively [4]. As OSMF advances, fibrosis extends to deeper oral structures [5]. Involvement of the tongue may reduce mobility, while fibrosis of oral musculature decreases tissue flexibility [6]. Extension into the pharyngeal region can also occur, leading to dysphagia [7]. A less recognized manifestation of OSMF is its effect on hearing. Fibrosis can influence the Eustachian tube (ET), which connects the middle ear to the nasopharynx and maintains pressure balance and fluid drainage [8]. Resulting hearing impairment can significantly affect communication, social functioning and quality of life [9]. Untreated ET dysfunction may progress to conductive or even sensorineural hearing loss if inner ear structures are compromised [10]. Thus, identifying auditory involvement and integrating otological evaluation into patient care is essential for improving outcomes [11]. Because the ET maintains middle-ear pressure equilibrium and prevents infections, understanding how OSMF affects its function remains important [12]. However, limited research exists on ET dysfunction across clinical stages of OSMF or its relationship to disease severity. With the emergence of CBCT imaging, structural and volumetric analysis of the ET has become feasible. Therefore, it is of interest to assess morphometric and volumetric ET changes in OSMF patients using CBCT and correlate these findings with audiometric alterations across disease stages to better evaluate morbidity and determine more effective therapeutic strategies.

## Methodology:

This prospective case-control study was conducted in the department of Oral Medicine and Radiology for a period of 2 years, after obtaining approval from Institutional Research and Ethical Board (IREB/2024/OMR/04). Study included 48 patients aged 18-75 years with a clinical diagnosis of OSMF, who required CBVI full mouth or full maxilla scans for endodontic, orthodontic, ENT or oral surgery purpose, which constituted the study group and 29 healthy individuals above age of 18 years with no discernible lesion in the mucosa or any systemic conditions, who constituted the control group. Patients with developmental defect, fracture, malignancy involving head and neck region, connective tissue disorder, ear wax, hearing aid, middle ear infections, trauma to ear, nasopharyngeal mass, working in noise area, history of previous ear surgery and pregnant women were excluded from the study. Total 77 participants were enrolled for this study who were categorised based on functional staging according to More *et al.* [13]. All study patients were subjected to brief clinical evaluation, CBCT and audiometric analysis for functional assessment of Eustachian tube.

## Procedure for CBCT:

CBCT volume scans of all subjects were obtained by using the KODAK Carestream Dental CS 9600 imaging system & the imaging protocol was used with voxel size 180 micrometer, 18-19 seconds scan time and 90 Kilovolts, for the field of view (FOV) of 16 x15.

## Eustachian tube length and volume assessment:

Measurements were performed using ITK-SNAP 4.2. Scan slices (1 x 1 mm) were analysed, with lengths recorded in millimetres and volumes in cubic centimeters. Eustachian tube length was measured on axial sections by identifying three points: A (tympanic orifice), B (junction of bony and cartilaginous portions) and C (pharyngeal orifice). The A-B (bony ET) and B-C (cartilaginous ET) distances were summed to obtain total length (Figure 1 - see PDF). Volume was assessed on axial CBCT images at the level of the maxillary first molar root tip (FOV: 16 x 15). Radiolucency from the nasopharyngeal opening to the maximum detectable distance was included. Multiplanar and 3D reconstructions were used to refine measurements, aligning the axial plane to the upper first molar root tip (Figure 2 - see PDF).

## Audiometric technique and evaluation:

Audiological testing was conducted using an Interacoustics AD-629 audiometer. Pure-tone audiometry assessed air conduction via the external and middle ear pathways and bone conduction via mastoid vibration. Testing occurred in a sound-treated booth with ANSI-calibrated equipment. Results were plotted on an audiogram displaying frequency (Hz) and hearing level (dB HL). Hearing loss was classified as: 0-15 dB (normal), 16-25 dB (minimal), 26-40 dB (mild), 41-55 dB (moderate), 56-70 dB (moderate-severe), 71-90 dB (severe) and >90 dB (profound).

## Results:

The mean age of participants in study group was 41.90 ± 15.87 years and in control group was 44.86 ± 17.98 years, with majority of patients being male in both Study (75%) and control (69%) groups (p>0.05). Mouth opening measurements were categorized into four groups showed that 50% of the subjects had an interincisal opening between 25 and 35 mm (M2), followed by 22.9% with a mouth opening between 15 and 25 mm (M3), 14.6% with openings greater than or equal to 35 mm (M1) and 12.5% with less than 15 mm (M4). Similarly, the Oral Submucous Fibrosis (OSMF) stage distribution mirrored these findings, with 50% of the cases classified as M2, 22.9% as M3, 14.6% as M1 and 12.5% as M4 (Figure 3 - see PDF). The paired t-test results for control group showed no significant differences between the right and left ET lengths (p>0.05) and volumes (p>0.05). However, in study group, significant differences were found between the right and left lengths (p<0.05) and volumes (p<0.05) (Table 1). Furthermore, comparisons between study and control groups demonstrated significant differences in ET lengths and volumes (p<0.05) (Table 2). The Kruskal-Wallis test showed significant differences in ET lengths and volumes across different OSMF stages (M1, M2, M3, M4) at p<0.05 (Table 3). Similarly, the Mann-Whitney and Wilcoxon's tests confirmed significant differences in lengths and volumes between different OSMF grades at p<0.05 (Table 4). Audiometric assessment indicated that 54.2% of study group participants had normal hearing, whereas 35.4% exhibited minimal hearing loss, 4.2% mild and 6.3% moderate hearing loss, whereas all control group participants had normal hearing (Figure 4 - see PDF). The t-test comparing audiometric results between study group and control groups was significant (p<0.05), indicating a substantial difference in hearing function. Moreover, the chi-square test revealed a significant association between audiometry results and OSMF stages in the study group (p<0.05) (Table 5).

## Discussion: 

The mean age of the study population was 41.90 ± 15.87 years, indicating that OSMF predominantly affects middle-aged adults. The broad age range (18-74 years) reflects its presence across younger and older groups. Nearly half of the patients (45.8%) were aged 18-38 years, suggesting early adulthood onset likely associated with early exposure to areca nut chewing. These findings are consistent with Kumar *et al.* (2015) [14], who noted higher OSMF prevalence among younger adults due to habitual areca nut use. Males constituted 75% of the sample, likely due to cultural and behavioral patterns such as greater tobacco and areca nut consumption. This gender disparity aligns with earlier findings by Ranganathan *et al.* (2004) [15]. Mean ET lengths in the case group (28.75 mm right, 29.03 mm left) were significantly shorter than in controls (37.61 mm right, 38.07 mm left), likely due to fibrosis-induced structural restriction Pottam *et al.* [16] found no significant length differences between cases and controls, through lengths decreased with increasing disease severity. ET volume assessments showed significantly reduced volumes in cases (275.15 mm³ right, 278.81 mm^3^ left) compared with controls (342.69 mm^3^ right, 346.21 mm^3^ left), attributable to fibrotic narrowing of the lumen. Similar volumetric reductions were reported by Patel *et al.* [17]. However, Pottam *et al.* [16] observed no significant volume differences between groups, through volume decreased with higher grades, without right-left variation. These ET length and volume reductions highlight the anatomical impact of OSMF and its potential to cause ET dysfunction. CBCT and ITK-SNAP enabled precise measurement, consistent with methods used by Pottam *et al.* [16]. Paired t-test results showed no significant right-left differences in the control group, indicating symmetrical ET development. In contrast, OSMF cases showed significant asymmetry, likely reflecting uneven fibrosis. Patel *et al.* [17] similarly reported asymmetry in affected craniofacial structures. The Kruskal-Wallis test demonstrated significant differences in ET dimensions across OSMF stages, indicating progressive structural alteration. Mann-Whitney and Wilcoxon tests confirmed greater changes in advanced stages. Bishnoi *et al.* [18] likewise linked fibrosis severity with increasing anatomical distortion. Audiometric evaluation showed that 54.2% of OSMF cases had normal hearing, while 35.4% had minimal, 4.2% mild and 6.3% moderate hearing loss. All controls had normal hearing, indicating OSMF-related ET dysfunction may lead to conductive loss. Pandiar *et al.* (2023) [19] similarly highlighted impaired ET ventilation due to fibrosis. Hearing impairment increased significantly with advancing OSMF stages, as shown by the chi-square test, consistent with Singh *et al.* [11], who reported progressive conductive hearing loss. The fibrosis-induced rigidity of the ET compromises pressure equalization, underscoring the need for regular audiologic monitoring. These stage-wise differences highlight the clinical value of audiometry in assessing disease severity, especially in advanced cases. Bishnoi *et al.* [18] emphasized early identification and intervention for auditory complications. This study has limitations, including a modest sample size, single-institution design and potential selection bias. The cross-sectional nature precludes assessment of progressive changes. Although CBCT and audiometry provided detailed evaluations, additional diagnostic tools could strengthen future studies. Potential confounders, such as differences in disease severity and patient compliance, also warrant consideration. Larger, multi-center longitudinal research is recommended to validate these findings.

## Conclusion:

OSMF causes significant reduction in eustachian tube length and volume, leading to measurable auditory impairment and reflecting its broader pathological impact on craniofacial structures. Data shows the importance of early diagnosis and comprehensive interdisciplinary management involving dental, ENT and audiology specialists. Further large-scale longitudinal studies are required to clarify long-term outcomes and guide effective interventions for ET dysfunction in OSMF patients.

## Figures and Tables

**Table 1:** ET Length and ET volume in study participants

**Descriptive statistics**		**Study group**	**Control group**
ET Length on Right side	Mean±S.D.	28.75±4.03	37.6103±1.89
(in mm)	Range	19.50-36.00	33-42
ET Length on Left side	Mean±S.D.	29.03±3.92	38.0724±1.78
(in mm)	Range	19-37	34-41
ET volume on Right side	Mean±S.D.	275±23	343±28
(in mm3)	Range	218-318	299-399
ET volume on Left side	Mean±S.D.	279±21	346±31
(in mm3)	Range	216-320	278-398

**Table 2:** Comparison of ET Length and ET volume in between case group and control group

**Paired Samples Test**						
**Variable**		**Paired Differences**			**T-value**	**p-value**
		**Std. Error Mean**	**95% Confidence Interval of the Difference**			
			**Lower**	**Upper**		
Pair 1	Length right study group - lenth right control group	0.81862	-10.7045	-7.35071	11.028	0.001**
Pair 2	Length left study group - length left control group	0.68974	-10.7784	-7.95264	13.578	0.001**
Pair 3	Volume right study group - volume right control group	5.77314	-79.5844	-55.9329	11.737	0.001**
Pair 4	Volume left study group - volume left control group	5.62856	-81.4261	-58.367	12.418	0.001**

**Table 3:** Comparison of different functional grades of OSMF (M1, M2, M3 and M4) with ET length and ET volume by Kruskal-Wallis test

**VARIABLE**	**OSMF STAGE**	**N**	**Mean Rank**	**CHI-VALUE**	**P-VALUE**
ET length on right side-	M1	7	42	35.365	0.001
Study group	M2	24	29.83		
	M3	11	13.18		
	M4	6	3.5		
	Total	48			
ET length on left side- study group	M1	7	44.43	33.342	0.001
	M2	24	27.9		
	M3	11	15.86		
	M4	6	3.5		
	Total	48			
ET volume on	M1	7	41.29	31.857	0.001
Right side- study group	M2	24	29.54		
	M3	11	13.41		
	M4	6	5.08		
	Total	48			
ET volume on	M1	7	42.14	29.194	0.002
Left side- study group	M2	24	28.44		
	M3	11	14.95		
	M4	6	5.67		
	Total	48			

**Table 4:** Comparison of Different Functional Grades of OSMF (M1, M2, M3 and M4) with ET Length and ET Volume by Mann Whitney Test and Wilcoxon’s Test

**Study group variables**	**OSMF stage**	**N**	**Mean rank**	**Sum of ranks**	**Mann-whitney U value**	**Wilcoxon’s Z value **	**P-value**
ET length on right side	M1	7	25	175	21.002	3.147	0.003**
	M2	24	13.38	321			
	M3	11	12	132			
	M4	6	3.5	21			
ET length on left side	M1	7	27.43	192	5.482	3.806	0.001**
	M2	24	12.67	304			
	M3	11	12	132			
	M4	6	3.5	21			
ET volume on	M1	7	24.29	170	26.457	2.776	0.003**
Right side	M2	24	13.58	326			
	M3	11	11.68	128.5			
	M4	6	4.08	24.5			
ET volume on	M1	7	25.14	176	20.412	3.047	0.002**
Left side	M2	24	13.33	320			
	M3	11	11.36	125			
	M4	6	4.67	28			

**Table 5:** Comparison of pure tone audiometric results between study group and control group

	**Paired Differences**		**T-value**	**p-value**
	**Std. Error Mean**	**95% Confidence Interval of the Difference**			
		**Lower**	**Upper**		
Audiometry study group-audiometry control group	0.14484	0.2895	0.883	4.047	0.001
